# Translational Regulation by eIFs and RNA Modifications in Cancer

**DOI:** 10.3390/genes13112050

**Published:** 2022-11-06

**Authors:** Linzhu Zhang, Yaguang Zhang, Su Zhang, Lei Qiu, Yang Zhang, Ying Zhou, Junhong Han, Jiang Xie

**Affiliations:** 1 School of Basic Medical Sciences, Chengdu University of Traditional Chinese Medicine, Chengdu 611137, China; 2 The Third People’s Hospital of Chengdu, Clinical College of Southwest Jiao Tong University, Chengdu 610014, China; 3 State Key Laboratory of Biotherapy, Frontiers Science Center for Disease-Related Molecular Network and Cancer Center, West China Hospital, Sichuan University, Chengdu 610041, China

**Keywords:** translation initiation factor, RNA modification, tumor, N6-methyladenosine (m^6^A), N6, 2′-O-dimethyladenosine (m^6^Am), 5-methylcytosine (m^5^C), pseudouridine (Ψ), N4-acetylcytidine (ac^4^C)

## Abstract

Translation is a fundamental process in all living organisms that involves the decoding of genetic information in mRNA by ribosomes and translation factors. The dysregulation of mRNA translation is a common feature of tumorigenesis. Protein expression reflects the total outcome of multiple regulatory mechanisms that change the metabolism of mRNA pathways from synthesis to degradation. Accumulated evidence has clarified the role of an increasing amount of mRNA modifications at each phase of the pathway, resulting in translational output. Translation machinery is directly affected by mRNA modifications, influencing translation initiation, elongation, and termination or altering mRNA abundance and subcellular localization. In this review, we focus on the translation initiation factors associated with cancer as well as several important RNA modifications, for which we describe their association with cancer.

## 1. Introduction

Translation is an essential step in the regulation of gene expression. Cells consume a large amount of energy in the process of mRNA translation and protein synthesis [1]. In eukaryotes, translation initiation is a complex and highly regulated process, and this regulation is necessary for many important life processes, requiring the participation of at least a dozen protein factors [2]. At the same time, eukaryotic initiation factors (eIFs) regulate the translation of mRNA, which is crucial for gene expression. This regulation primarily occurs at the initiation stage of eukaryotic translation, which is the rate-limiting step of protein synthesis. The dysregulation of mRNA translation is a collective characteristic of tumorigenesis, and aberrant changes in the components of the translational machinery are also frequently found in cancer. For instance, the atypical behavior of the eIF complex activated by upstream signaling pathways has been detected in non-cancer-related human diseases and numerous tumors, resulting in the selective expression of the encoded proteins associated with tumorigenesis, metastasis, or anti-tumor drug resistance [3,4]. The translational regulation of pre-existing mRNA is more direct, faster, and more powerful than transcriptional regulation. It sensitively alters the levels of encoded proteins in cells, thereby changing the expression of the proteome rapidly without requiring alterations in RNA synthesis to allow cells to adapt to physiological and pathological conditions. The translation of eukaryotic genes is a very complex and precise process. The four main stages include initiation, elongation, termination, and the recycling of ribosomes [5].

Over the last five decades, RNA modifications have been identified using a variety of methods. However, it was only in the last decade that eukaryotic RNA modifications were identified and characterized, primarily in transfer RNAs (tRNAs) and ribosomal RNAs (rRNAs), followed by mRNAs and various noncoding RNAs [6]. The specific chemical modification of mRNA is an effective way to modulate molecular function, and the modification of mRNA and proteins can alter downstream signaling pathways. Therefore, in recent years, the study of RNA epitope transcriptomics has emerged as a promising new area in the field of RNA modification. The dysregulation of RNA modification pathways has also been found in human cancers, which suggests that they may be ideal targets for cancer treatment [7]. Global biological macromolecules (RNAs, DNAs, sugars, lipids, and proteins) undergo synthetic and covalent modifications. The widest variety of modifications has been found on proteins and RNA in animal cells. Over the past 50 years, more than one hundred different types of post-transcriptional RNA modifications have been identified using various methods [8]. With the deepening of research, it became clear that many molecular processes are regulated by precise RNA modifications, including RNA decay, splicing, localization, translation, stability, and metabolism, thus maintaining the diversity of genetic information. The epigenetic modification of RNA is mediated by three distinct classes of proteins that specialize in “writing” (catalyzing the deposition of specific modifications), “erasing” (catalyzing the removal of specific modifications), and “reading” (recognizing and binding specifically modified nucleotides), thereby affecting the fate of RNA [9]. The modification of RNA is a dynamic process in the body that allows cells to respond rapidly to environmental signals at any time. Adapting to a changing microenvironment, for instance, caused by stress or chemotherapeutic drug treatment, is essential for cancer cell development, which suggests that RNA modifications may play a key role in tumor progression [10,11]. Historically, cancer itself has also been a disease characterized by epigenetic or genetic alterations in tumor suppressor genes and oncogenes [12]. Although RNA modifications are sometimes not deemed to be cancer-initiating factors, numerous studies have illustrated that aberrant RNA modifications are often involved in functions affecting cell self-renewal, survival, proliferation, invasion, differentiation, and chemoresistance, all of which are associated with the characteristics of cancer [13,14,15].

**Figure 1:**
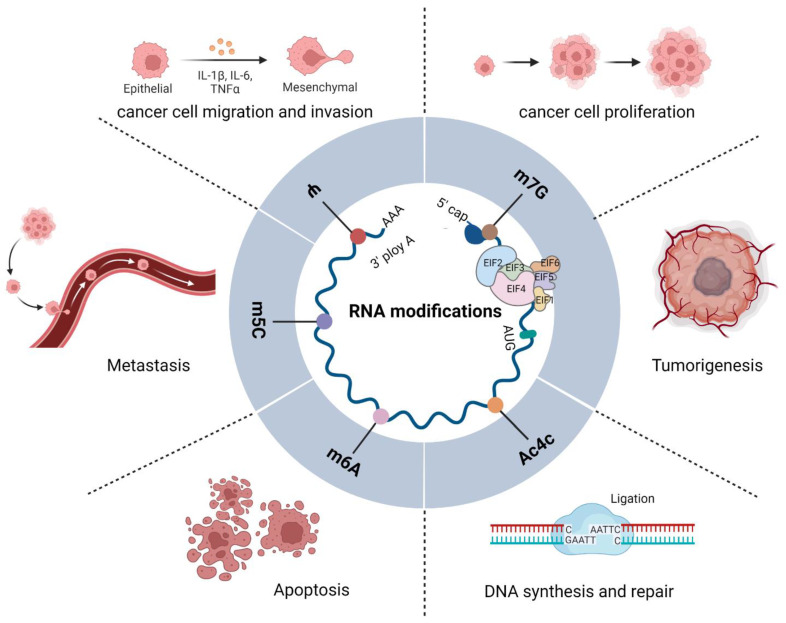
Functions of eIFs and RNA modifications in tumors. Diagram showing the functions of eIFs and m^6^A, m^6^Am, m^5^C, Ψ, and ac^4^C in RNA modification. A variety of eIFs constitute translation initiation complexes that regulate RNA modification and cellular protein translation, directly or indirectly; they interact with one another to regulate the occurrence, development, and biological behavior of tumors.

In this review, we examine the present understanding of the fundamental function of eIFs in translation initiation, with a specific discussion of the dysregulation of eIFs and their roles in the initiation of mRNA translation as well as in tumorigenesis and metastasis (Figure 1). In addition, we describe the cellular and molecular functions of RNA modifications in regulating gene expression programs, focusing on N6-methyladenosine (m^6^A), N6,2′-O-dimethyladenosine (m^6^Am), 5-methylcytosine (m^5^C), pseudouridine (Ψ), and N4-acetylcytidine (ac^4^C) and their roles in cancer physiopathology (Figure 1). In addition, we provide insights into the mechanisms of these post-transcriptional modifications affecting tumor progression and maintenance.

## 2. Overview of eIFs in Translation Initiation

**Figure 2:**
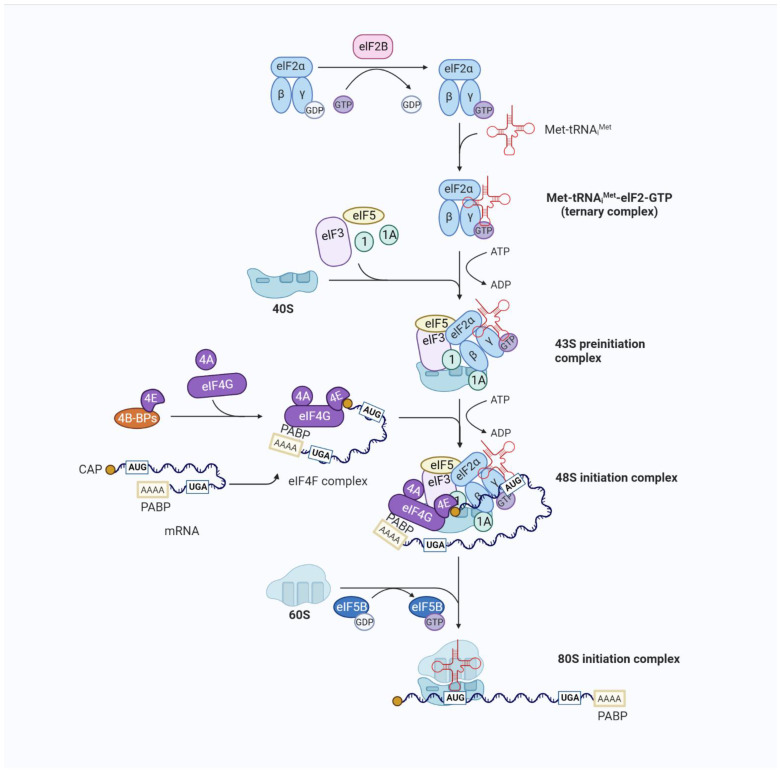
Overview of eukaryotic translation initiation. Translation is an iterative process. The typical mechanism for the regulation of translation occurs at the rate-limiting stage of initiation, starting with the formation of the EIF2/GTP/Met-tRNA_i_^Met^ TC and involving the control of the functional 40S subunit and its related factors (the 43S preinitiation complex (43S PIC)) assembly. The 43S PIC is a large complex formed by the binding of the 40S ribosomal subunit to the eukaryotic translation initiation factors eIF1, eIF1A, eIF3, and eIF5 and the TC. The recruitment of the 43S PIC to the mRNA template is facilitated by eIF4F, a complex consisting of the mRNA 5′-cap-binding subunit (eIF4E), a large scaffold protein (eIF4G), and an RNA helicase (eIF4A), resulting in the 48S PIC form. eIF4F recruits ribosomes to mRNA through the eIF4E-mRNA cap and eIF4G-eIF3 interaction to form the 48S initiation complex. eIF4G also interacts with poly(A)-binding protein (PABP), which binds to the mRNA 3′ poly(A) tail, resulting in mRNA circularization to stabilize mRNA and facilitate translation. The eIF4A helicase, which unfolds structures near the mRNA 5′-cap with other stronger helicases (DHX29), is involved in the initial interaction of eIF4F with the 5′ end of mRNA and can also facilitate the scanning of the 40S ribosomal subunit towards the start codon by resolving secondary structures in the 5′ untranslated region (UTR). The recognition of the initiation codon by the 43S PIC results in the release of eIF and the joining of the 60S subunit. The formation of the translationally competent 80S ribosome marks the end of initiation and the beginning of elongation.

Translation initiation is a complex process during which eIFs assemble the initiator tRNA (Met-tRNA_i_^Met^) and the 40S and 60S ribosomal subunits into 80S ribosomes at the mRNA initiation codon. Among them, Met-tRNA_i_^Met^ is located at the ribosomal P site of the initiation codon [16]. The complex initiation process of eIF-mediated 80S ribosome formation involves four stages [5] (Figure 2). First, eukaryotic initiation factor 2 (eIF2) selects Met-tRNA_i_^Met^ from the extended tRNA pool and combines the eIF2/GTP/Met-tRNA_i_^Met^ ternary complex (TC) and other eIFs with the 40S subunit to form a 43S preinitiation complex. Second, the eukaryotic initiation factor 4 subunit E (eIF4E) (cap binding) and F(eIF4F) initially recognize the N7-methylguanosine (m^7^G) cap at the 5′-terminus, allowing the 43S complex to bind to the mRNA. Then, the mRNA-bound ribosome complex moves along the 5′ untranslated region (5′UTR) from its initiation binding site to the initiation codon, forming a 48S initiation complex. The initiation codon pairs with the promoter anticodon of tRNA in this 48S initiation complex. Finally, the displacement of factors from the 48S complex and the joining of the 60S subunit to form an 80S ribosome leaves Met-tRNAiMet in the ribosomal P site. This facilitates the synthesis of the first peptide bond, and the process continues to the translational elongation stage. In eukaryotes, translation initiation culminates with formation of an 80S initiation complex in which Met-tRNAi Met is bound in the P site of the ribosome. Translation and translational regulation are considered key links in the adaptation of the tumor stress response to overcome various conditions, such as continuous proliferation, immune surveillance, the tumor microenvironment, and the cytotoxicity of drugs [17].

Disorders in mRNA translation play a significant role in the pathogenesis of malignant tumors. Abnormal translation pathways promote tumor growth and cell transformation. In normal cells, aberrant translational pathways affect energy, stress, nutrient supply, gene expression, and ribosome production; however, in some cells they are overactivated and promote cancer [18,19]. Eukaryotes have evolved a complex translation initiation pathway. There are at least 12 dedicated proteins, the eIFs, that each play critical roles in the process, and several of these factors are comprised of multi-subunit proteins. A host of eIFs are protein complexes composed of a few subunits. The dysregulation of their expression through phosphorylation, downregulation, or overexpression leads to various types of carcinogenic progression [17,20,21,22]. Table 1 lists the functions of eIFs and their associated subunits in translation initiation.

### 2.1. eIF1 and Tumors

Eukaryotic initiation factor 1 (eIF1) and eukaryotic initiation factor 1 subunit A (eIF1A) are essential to facilitate the initiation of the translational scanning process. Research has confirmed that when both eIF1 and eIF1A bind to 40S, the open conformation of the “latch” involving the mRNA entry channel undergoes structural rearrangement, allowing the PIC to scan. By contrast, the 40S/eIF1A complex may prevent scanning by leaving the “latch” in a locked conformation after eIF1 release. Importantly, eIF1 and eIF1A together stimulate the speed of TC binding to 40S and accurate AUG recognition; in the absence of eIF1, TC binds more tightly to the PIC, resulting in a sub-stable state of the TC that facilitates scanning but is incompatible with start codon recognition and binds to the “open” conformation of the PIC. Upon AUG recognition, the TC achieves a more stable interaction with the P-site by the isomerization reaction, which requires eIF1 dissociation and subsequent rearrangement to a closed 40S conformation [38,39]. Recently, it was discovered that somatic mutations in eIF1A, especially the N-terminal tail, are related to uveal cancer, thyroid cancer, and ovarian cancer [40,41,42,43].

Cancer-related N-terminal tail mutants reduce the interaction between eIF1A and the A-site ribosomal scanning arrest proteins Rps10 and Rps3 [44]. The presence of eIF1A mutations has also been reported in diverse types of melanoma and epithelial carcinoma [41,42]. However, further research is needed to reveal the biological functions of eIF1A in cells and how these functions contribute to cancer.

### 2.2. eIF2 and Tumors

eIF2 is a heterotrimeric initiation factor composed of eIF2α, β, and γ subunits, which together participate in the formation of the eIF2–Met-tRNA_i_^Met^–GTP complex. During initiation, the GTP in the TC is hydrolyzed to GDP, and eIF2–GDP must be recycled to eIF2–GTP for renewed TC assembly, a reaction catalyzed by the heteropentameric eIF2B complex, in particular the carboxy-terminal segment of eIF2Bε, which interacts directly with the G domain of eIF2γ and the lysine-rich regions of eIF2β. The other eIF2B subunits also contribute to eIF2–GDP binding through interactions with eIF2α; these interactions are enhanced by the phosphorylation of Ser51 by one of the eIF2α kinases, which are activated in stress conditions to downregulate general initiation. Meanwhile, the recycling of eIF2–GDP by eIF2B is negatively regulated by the formation of a competing eIF5–eIF2–GDP complex [45,46,47].

Currently, 29 eIF2B mutations have been reported, all of which render eIF2B insensitive to eIF2 phosphorylation [45]. In addition, the catalytic subunit of eIF2β is upregulated in diverse cancers and is related to tumorigenesis. The inhibition of this subunit in vitro can slow tumor progression; therefore, eIF2β may be an ideal therapeutic target for cancer [48]. One study found that the deletion of eIF2β led to G1 arrest in lung cancer cells, and the authors speculated that eIF2β knockout might have done so by reducing the binding of GTP to eIF2; however, more in-depth research is needed to support this hypothesis [48]. The translation of activating transcription factor 4 is promoted by phosphorylated eIF2α and is involved in osteoblast differentiation and bone formation [49,50]. Furthermore, eIF2α has been reported to be elevated in transformed lymphocytes from patients with non-Hodgkin’s lymphoma; this phenomenon was not observed in the mantle zones and surrounding paracortices, demonstrating that eIF2α expression was positively correlated with lymphocyte proliferation [51]. The aggressiveness of various lymphoma subtypes is positively linked to the expression level of eIF2 [52]. Collectively, eIF2 plays a significant role in protein synthesis, ribosomal energy metabolism, the cancer cell cycle, tumorigenesis, and development.

### 2.3. eIF3 and Tumors

eIF3 is the most complex and largest initiation factor in the translation initiation complex (TIC) with 13 subunits, eIF3a–m. They can be assembled into different eIF3 complexes. Among these subunits, eIF3a, b, c, e, f, and h are core components of the eIF3 complex [53]. eIF3 contributes to the hyperactivation of the translation initiation machinery and thereby plays a significant role in tumorigenesis [54]. The overexpression of eIF3 complex subunits, including eIF3a, b, c, h, and i, promotes the malignant transformation of fibroblasts by stimulating global protein synthesis and the translation of specific mRNA transcripts [55].

eIF3a, c, and i are overexpressed in a variety of cancers [56]. Ge Li et al. found that eIF3a not only promoted the expression of c-myc and cyclin D1 proteins but also blocked the phosphorylation of Raf-1 and ERK and affected the cell differentiation and morphological maturation of chronic myeloid leukemia cells [57]. eIF3c may serve as a direct target of YTHDF1, the “reader” of m^6^A. YTHDF1 enhances the translation of eIF3c in an m^6^A-dependent manner by binding to eIF3c mRNA, thereby promoting tumorigenesis and metastasis in ovarian cancer [58]. In addition, eIF3i upregulates COX-2 synthesis to regulate Wnt/β-catenin signaling and integrates with the PI3K/mTOR signaling pathway to induce abnormal colorectal cancer (CRC) proliferation and tumorigenesis [59]. According to molecular evidence, eIF3d may slow the progression of prostate cancer by regulating the translation of proteins involved in multiple signaling pathways, including those associated with the cell cycle and drug responses [60].

eIF3h is highly expressed in prostate and breast cancer. Interestingly, the expression of eIF3h was positively associated with poorly differentiated and aggressive prostate cancer [61]. Likewise, a high expression level of eIF3h was related to the migration, proliferation, and invasion of human hepatocellular carcinoma [62]. The amplification of the *eIF3h* gene was found in non-small cell lung cancer and CRC by fluorescence in situ hybridization and genome-wide analysis, respectively [63,64]. The *eIF3-p48* gene was identified for the first time as a common integration site of murine mammary tumor virus in murine mammary tumors. The *eIF3-p48* mutation observed in murine mammary tumor virus-induced tumors and hyperplasia contributes to the malignant transformation of mammary epithelial cells [65]. It has been reported that eIF3e expression was reduced in 31 percent of non-small-cell lung cancer patients and 37 percent of breast cancer patients. In contrast, the high expression of wild-type eIF3e does not promote malignant transformation, suggesting that wild-type eIF3e may have a tumor suppressor effect while truncated eIF3e has oncogenic potential [66].

eIF3f is the only eIF3 subunit that is downregulated in pancreatic cancer and melanoma. eIF3f induces apoptosis by inhibiting protein synthesis and proliferation in pancreatic cancer and melanoma cells, whereas eIF3f may act as a negative regulator of translational effects, and its low expression protects melanoma cells from apoptosis [67]. In fact, decreased eIF3f mRNA transcript levels are commonly detected in tumors such as breast cancer, ovarian cancer, melanoma, and pancreatic cancer [68]. Taken together, eIF3e and eIF3f may act as negative regulators of mRNA translation.

### 2.4. eIF4 and Tumors

The eIF4 complex has multiple components that can recruit ribosomal subunits to mRNA [69]. Mammalian eIF4B can stimulate the helicase activity of eIF4A, an activity it shares with its homolog, eIF4H. In addition, the binding and scanning of mRNA transcripts by the PIC in vitro is highly dependent on eIF4B [70,71]. Another study showed that eIF4B increased the efficiency with which eIF4G stimulated ATP hydrolysis coupled to RNA duplex unwinding by eIF4A, while eIF4H was less efficient in this regard. This is because eIF4H is shorter and lacks most of the carboxy-terminal region of eIF4B [72]. eIF4A plays an even more important role when it is mapped as a molecular nexus downstream of critical oncogenic signaling pathways (RAS, PI3K/AKT/mTOR, and MYC). It provides a direct link between the necessary steps in lymphomagenesis metastasis and the scanning of the 43S preinitiation complex [73,74].

At present, the function of eIF4B has not been fully elucidated. eIF4B binds to eIF4F to form a complex and regulates the activity of eIF4F, and it is considered unimportant for translation initiation [17]. Previous studies have shown that during translation initiation, eIF4B stimulates eIF4A activity by regulating the eIF4A conformational cycle and binding to the eIF3 complex through the eIF3A subunit [72,75]. In addition, eIF4B can be phosphorylated by activated S6K kinase, thereby enhancing the interaction between eIF4B and eIF3A, which is a downstream target of mTOR [76]. Targeting eIF4B suppresses the chemoresistant and metastatic properties of non-small cell lung cancer [77]. eIF4B has been shown to ensure cell survival by specifically promoting the translation of genes associated with cell proliferation as well as survival [78].

eIF4E is a cap-binding protein that specifically recognizes the 5′ m7G cap through stacking interactions with two tryptophan residues between two conserved tryptophans found in the cap-binding pocket of eIF4E. Meanwhile, eIF4E plays a key role in mRNA recruitment to control translation initiation and the translation rate [17]. In addition, *eIF4E* acts directly as an oncogene in vivo. Transgenic mice overexpressing eIF4E will experience increased tumorigenicity and resist treatment with drugs [79]. Numerous studies have elucidated that eIF4E and phosphorylated eIF4E are present in various types of cancer. The overexpression of eIF4E has been found in approximately 30% of human cancer cases [80,81]. MNK1 and MNK2 are recruited by the eIF4G protein of eIF4E to phosphorylate eIF4E, resulting in uncontrolled translation and the inhibition of apoptosis and proliferation [82,83]. eIF4E serves as the subunit of the eIF4F complex, and its ability to build a cap-binding eIF4F complex is regulated by 4E-binding protein 1 (4E-BP1). In addition, the phosphorylation of eIF4E contributes to its interaction with 4E-BP1 [84,85]. The activation of the mTOR pathway by the phosphorylation of 4E-BP1 reduces the binding of eIF4E to 4E-BP1, resulting in eIF4E hyperactivation [86].

When eIF4E interacts with the eIF4F complex, the hyperactivation of eIF4E results in translation initiation. Thus, the hyperphosphorylation of 4E-BP1 is connected with CRC [87], breast cancer [88], ovarian cancer [89], prostate cancer [90], lung cancer [91], lymphoma [92], and head and neck cancers [93]. In vivo, the high expression of eIF4E resulted in lung cancer, lymphoma, and angiosarcoma in transgenic mice [79]. Disrupting the interaction of eIF4E with the cap may be a possible anti-tumor approach, and researchers have conducted experiments to achieve this goal [94]. Likewise, in vitro studies reported that breast cancer cell migratory activity and invasiveness were significantly attenuated when eIF4E was knocked out [95].

eIF4G is a scaffold protein of the TIC, responsible for recruiting ribosomal subunits, mRNA, and cap-binding complexes to assemble in order to accomplish translation. eIF4G enhances the association of eIF4E with the cap structure through an allosteric enhancement mechanism [96]. eIF4G has binding sites for eIF4E [96], eIF4A [72], and eIF3a through an N-terminal poly(A)-binding protein (PABP) site [18,21]. Studies have shown that the inhibition of eIF4G/eIF4E complex interaction by the translation initiation inhibitor 4EGI-1 leads to growth restriction in human melanoma and breast cancer cells [97].

eIF4H is a cofactor of eIF4A. At the same time, eIF4H and eIF4B have similar abilities to stimulate eIF4A’s helicase activity; however, the stimulation by eIF4B is more pronounced in the presence of eIF4G. eIF4H and eIF4B bind to the same domain of eIF4A, and this binding is enhanced in the presence of ATP [98]. Studies have found that the dysregulation of eIF4A expression is associated with malignant transformation and is highly expressed in liver cancer and melanoma [99,100].

### 2.5. eIF5 and Tumors

eIF5 is one of the most important proteins in the translation initiation pathway and possesses two functions: at the N-terminus, it acts as a GTPase-activating protein (GTP hydrolyzation by eIF2) and an independent GDP dissociation inhibitor (through the controlled recycling of eIF2) [101,102]. At the same time, eIF5 may also inhibit the guanine nucleotide exchange factor eIF2B, which promotes eIF2 recycling [36]. eIF1 interacts with eIF5 and promotes the specific recognition of initiation codons. Histone deacetylase 2 can regulate the expression of eIF5 in lung cancer, and its high expression is associated with a poorer prognosis in lung cancer patients [103].

*eIF5A* is the gene encoding the eIF5 protein, which has extensive functions in translation elongation as well as termination; the deletion of eIF5A results in an overall translation elongation defect [104]. In mammals, eIF5 is the only known protein that undergoes post-translational hypusination, which is the incorporation of the rare amino acid hypusine [105]. The difference between eIF5A1 and eIF5A2 is that the former is expressed in all cells and is proportional to the rate of cell proliferation, while the latter is expressed in a tissue-specific manner and is almost undetectable in most cases [106]. Studies have shown that eIF5A2 increased resistance to doxorubicin by regulating the epithelial–mesenchymal transition of CRC. Thus, eIF5A2 may be an ideal potential target molecule for the treatment of CRC in the future [107].

eIF5B facilitates 60S ribosomal subunit ligation and can indirectly support the association of Met-tRNA_i_^Met^ with the ribosome during translation initiation. eIF5B interacts with eIF5, eIF2A, and eIF1A and indirectly supports the binding of Met-tRNA_i_^Met^ to ribosomes in translation initiation [108,109]. eIF5B is overexpressed in a variety of malignancies, and the abnormal expression of eIF5B is associated with glioblastoma [110], lung cancer [111], and hepatocellular carcinoma [112].

## 3. RNA Modifications

More recently, RNA modifications have been recognized as key post-transcriptional regulators of gene expression in eukaryotic cells. RNA modifications regulate multiple biological developmental pathways. The correct deposition of these modifications is essential for normal cellular development. Alterations in their deposition have been linked to several diseases, including cancer. Therefore, we concentrate on describing the presence of m^6^A, m^6^Am, m^5^C, Ψ, and ac^4^C and their pathophysiologic roles in tumors. In addition, we draw attention to recent insights into the mechanisms by which these post-transcriptional epigenetic modifications influence tumorigenesis and tumor progression.

### 3.1. m^6^A in Cancer

**Figure 3:**
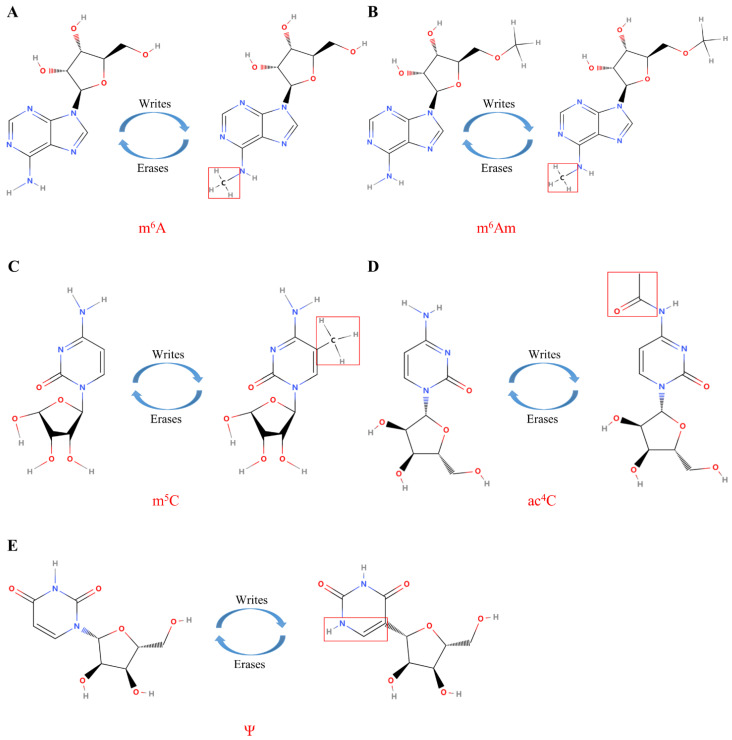
The molecular composition of RNA modifications. (**A**) The m^6^A modification of RNA is mediated by proteins called “writes” and “erases”; (**B**) The m^6^Am modification of RNA is mediated by proteins called “writes” and “erases”; (**C**) The m^5^C modification of RNA is mediated by proteins called “writes” and “erases”; (**D**) The ac^4^C modification of RNA is mediated by proteins called “writes” and “erases”; (**E**) The Ψ modification of RNA is mediated by proteins called “writes” and “erases”. Under the catalysis of the corresponding enzymes, the molecular structure and the enrichment level of RNA are changed to play different biological roles.

Among the many RNA modifications, the m^6^A modification is the most abundant and classical mRNA modification, which controls RNA translation, stability, and splicing. Simultaneously, m^6^A modification plays an important role in cell metabolism, differentiation, and growth. RNA can be dynamically and reversibly modified under the action of different types of enzymes; m^6^A interacts with “readers” (m^6^A-binding proteins), is catalyzed by “writers” (RNA methyltransferases), and is removed by “erasers” (demethylases) (Figure 3A). As more advanced techniques are employed in research, the profound molecular mechanisms of m^6^A RNA methyltransferases, demethylases, and binding proteins are beginning to be revealed. Furthermore, altered m^6^A RNA modification homeostasis has been associated with cancer [113]. The main roles of m^6^A writers, readers, and erasers in cancer are summarized in Table 2.

#### 3.1.1. m^6^A Writers in Cancer

A previous study showed that METTL3 was overexpressed in acute myeloid leukemia, in which it methylated BCL2, PTEN, and c-Myc mRNAs, leading to boosted translation and the inhibition of cell apoptosis and differentiation, thereby promoting leukemia progression [114]. In addition, another study confirmed that METTL3 could maintain acute myeloid leukemia cells in an undifferentiated state in vivo, thereby preventing the occurrence and development of myeloid leukemia [159]. In contrast, METTL14 acts as an oncogene in acute myeloid leukemia by augmenting the stability and translation of the *MYB* and *c-Myc* genes [115]. METTL3 promotes the translation of EGFR and TAZ, leading to lung and colon cancer cell growth, survival, and invasion [123]. METTL3 increases the methylation and stability of HBXIP mRNA by inhibiting the expression of the tumor suppressor gene *let-7g* in breast cancer, thereby inducing cell proliferation and survival [160]. Recent research studies have indicated that the abnormal accumulation of m^6^A has also been found in CRC. The aberrant deposition of m^6^A increases the expression of miRNA-1246, leading to the downregulation of the *SPRED2* tumor suppressor gene and the induction of metastasis [118]. A more recent finding conveyed that METTL3 was highly expressed in prostate cancer and promoted cancer cell growth and invasion through SHH-GLI1 signaling [127]. Moreover, METTL3 was also found to control the expression of ITGB1, which affects ITGB1 binding to type I collagen and promotes bone metastasis in prostate cancer [128].

In hepatocellular carcinoma and glioblastoma, several reports have revealed the consistent oncogenic roles of METTL3 and METTL14 in these two cancer types. However, some reports have demonstrated the tumor-suppressing effects of both. Preliminary studies have shown that m^6^A methylation inhibits the self-renewal, progression, and tumorigenesis of glioblastoma stem cells by reducing the stability and expression of major *ADAM19* oncogenic transcripts [12]. Meanwhile, another group of researchers found that METTL3 upregulation was observed to predict poorer patient survival and increased SOX2 mRNA expression and stability, promoting the growth of glioblastoma stem cells and shortening the survival time of mice [118]. Likewise, the m^6^A “writers” can facilitate or inhibit the occurrence and development of hepatocellular carcinoma. For example, METTL14 was originally considered as a tumor suppressor in liver cancer; METTL14 induced the increased expression of pri-miR-126, which inhibited tumor metastasis [161]. Conversely, Chen et al. discovered that METTL3 was upregulated in hepatocellular carcinoma patients and was correlated with worse prognosis due to its suppression of cytokine signaling-2 mRNA expression in an m^6^A-YTHDF2-dependent manner [121]. From the above reports, it can be concluded that the expression levels of m^6^A “writers”, “erasers”, and “readers” can account for the differences in m^6^A-targeted RNAs and in deposition, which lead to epigenetic differences. More detailed studies are needed, however, to characterize METTL3 and METTL14 in glioblastoma and hepatocellular carcinoma.

In endometrial cancer, 70% of tumors result in reduced m^6^A levels through the downregulation of METTL3 expression or METTL14 mutation. The loss of METTL3 and METTL14 complexes can upregulate the expression of AKT pathway members and lead to increased cell proliferation, which in turn exhibits tumor suppressor functions in endometrial cancer [162]. Likewise, the deletion of METTL3 in renal cancer promotes cell proliferation through the PI3K-AKT-mTOR pathway and activates the epithelial–mesenchymal transition pathway to promote cell invasion and migration [130].

Recently, some studies have been conducted on the role of METTL16 in cancer. In recent reports, changes in the expression, mutation, and loss of function of METTL16 all affected the occurrence and progression of CRC [163]. Other studies have reported that METTL16 is associated with the maturation of oncogene and tumor suppressor MALAT1 mRNA [164]. METTL16 regulates small nuclear RNA (snRNA) methylation and promotes tumor development by inducing changes in alternative splicing [165].

#### 3.1.2. m^6^A Erasers in Cancer

The dysfunction of m^6^A erasers has been implicated in the development of cancer. In fact, fat mass and obesity-associated gene (FTO) nucleotide polymorphisms are associated with an increased risk of cancer and a variety of human diseases [166]. Likewise, the inhibition of FTO has been shown to prevent GSC growth, self-renewal, and tumorigenesis; FTO is associated with poor survival due to its regulation of ADAM19, a gene with key biological functions in glioblastoma stem cells [12]. FTO expression was also shown to be increased in other tumors. For example, in melanoma, FTO expression reduces the methylation of SOX10, CXCR4, and PD-1 by m^6^A in key tumor-promoting melanoma cell-intrinsic genes to promote tumorigenesis [143]. In cervical cancer, FTO interacts with E2F1 and MYC to increase its translation efficiency and promote cervical cancer cell migration and proliferation [140]. In lung cancer, FTO was shown to be related to poor prognosis due to its promotion of cell invasion and proliferation by controlling MZF1 expression and inhibition of lung cancer cell apoptosis [142]. Additionally, in breast cancer, the overexpression of FTO promotes cell proliferation and metastasis in vivo by downregulating BNIP3 expression [139].

ALKBH5, the second m^6^A demethylase, is overexpressed in several cancer types [146]. The expression of ALKBH5 is correlated with poor prognosis in glioblastoma and promotes the proliferation and tumor progression of glioblastoma stem cells by enhancing FOXM1 expression [147]. ALKBH5 enhances breast cancer stem cell stability and promotes tumorigenesis by reducing methylation in KLF4 and NANOG mRNA [167]. In gastric cancer, ALKBH5 boosts metastasis and invasion by demethylating the ncRNA NEAT1 [148]. Increased ALKBH5 in ovarian cancer predicts poorer survival, as the high expression of ALKBH5 activates the mTOR and BLC2-Beclin1 pathways to promote proliferation, invasion, and even autophagy [168]. Similar to other m^6^A receptors, ALKBH5 acts as a tumor suppressor in a variety of tumors. In pancreatic cancer cells, the deletion of ALKBH5 increases the methylation of the long noncoding RNA (lncRNA) KCNK15-AS1, which negatively regulates its expression and enhances cell migration. ALKBH5 also inhibits tumorigenesis by decreasing WAF-1 expression levels and hampering the activation of Wnt signaling [152].

#### 3.1.3. m^6^A Readers in Cancer

In recent years, m^6^A “readers” have also been found to be abnormally expressed in tumors and associated with the development of cancer. Both YTH domain-containing 1 (YTHDC1) and YTHDF2 are overexpressed in CRC and are correlated with poor patient prognosis, metastatic potential, and cell proliferation. YTHDC2 controls the expression of the tumor-promoting gene *HIF1a* in these tumors [153]. The deletion of YTHDF1 inhibits the Wnt-β-catenin signaling pathway, thereby inhibiting tumorigenicity and tumor growth [154]. YTHDF1 is also upregulated in ovarian cancer. YTHDF1 upregulates the translation of eIF3c and promotes tumorigenesis and metastasis [58]. In contrast, YTHDF1 stimulates the translation of the tumor suppressor Hint2 in melanoma, thereby inhibiting tumor progression [155]. In acute myeloid leukemia, YTHDF2 can reduce the half-life of m^6^A-containing transcripts, thereby affecting tumor necrosis factor signaling and promoting apoptosis [156]. In addition, YTHDF2 was found to be abnormally overexpressed in lung cancer, where it promoted the translation of 6-phosphogluconate dehydrogenase to support tumor growth [158]. However, YTHDF2 was also able to inhibit cell proliferation and tumor growth and activate ERK and MEK signaling by inducing the degradation of EGFR mRNA in hepatoma cells [157].

### 3.2. m^6^Am Modifications in Cancer

m^6^Am was originally identified in viral mRNA and animal cells in the 1970s [169]. In contrast to m^6^A, which is an internal modification mainly present in the 3′ untranslated region (3′UTR), N6, 2′-O-dimethyladenosine (m^6^Am) contains one more methylated modification and is located adjacent to the cap structure of mRNA (Figure 3B). The first or second nucleotide near the m^7^G cap of the mRNA can be methylated at the 2′-hydroxyl. When the first nucleotide is 2′-O-methyladenosine (Am), it can be further methylated to m^6^Am at the N6 site under the catalysis of a specific methylase [170]. m^6^Am plays a crucial role in snRNA biogenesis [171], RNA splicing [172], mRNA cap-dependent translation, and stability [173,174].

Recent reports have shown that FTO, which is expressed at low levels in patient-derived cells, hinders the ability of cancer stem cells in CRC through its demethylase activity and increases m^6^Am levels in mRNA, resulting in enhanced tumorigenicity and chemoresistance in vivo [175] (Table 3). Past studies have acknowledged phosphorylated C-terminal domain-interacting factor 1 (PCIF1) as the only known m^6^Am “writer” protein; however, the additional catalytic functions of PCIF1 require further study [176]. The overexpression of m^6^Am methyltransferase PCIF1 in glioma blocks G0/G1 phase progression and induces glioma cell apoptosis, whereas the downregulation of PCIF1 promotes glioma cell proliferation [177]. Expression differences between normal tissues and tumors were obtained based on the Genotype Tissue Expression and Cancer Genome Atlas (TCGA) databases. It was found that the high expression of PCIF1 was negatively correlated with overall survival or disease-free survival in gastric cancer patients [178]. In addition, PCIF1 overexpression was found to promote the infiltration of CD4+ T cells in renal clear cell carcinoma and the infiltration of CD8+ T cells, macrophages, and B cells in thyroid cancer in vivo [179]. The role of m^6^Am in cancer development and progression has been newly proposed in recent years, and its specific mechanism remains to be elucidated by further research.

### 3.3. 5-Methylcytosine

m^5^C is a universal and conserved RNA modification that exists in all areas of life (Figure 3C). m^5^C is widely present in a variety of RNA transcripts, including cytoplasmic and mitochondrial rRNA and tRNA, as well as mRNA, enhancer RNA, and some noncoding RNAs, but not in eukaryotic tRNAs. Among these, rRNA was found to hold the highest content [180]. In eukaryotic cells, the C5 methylation of RNA cytosines is catalyzed by the DNA methyltransferase homolog DNMT2 and the NOL1/NOP2/SUN domain (NSUN) family of enzymes. Recent research has identified the exact target nucleotides for the modification of the corresponding substrate proteins and RNA transcripts of various methyltransferases. These studies have provided a deeper molecular understanding of the oncogenic or tumor-suppressive effects caused by mutations in genes encoding m^5^C methyltransferases or by changes in the expression levels of these enzymes [181] (Table 4).

#### 3.3.1. m^5^C Writers in Cancer

In past studies, cytosine-5 methyltransferases have been revealed to play an important role in several cancers. The high expression of NOP2 was found to promote the proliferation of mouse fibroblasts [209]. At the molecular level, NOP2 interacts with the T-cell factor of the cyclin D1 promoter in cancer cells, recruiting telomerase RNA component elements and activating cyclin D1 transcription. It has been found that NOP2 is highly expressed in breast cancer, lung cancer, prostate cancer, and gallbladder cancer, and its expression level is positively correlated with poor prognosis [183,184,210]. However, whether NOP2 is involved in the methylation activity of rRNA needs to be further elucidated [211]. Changes in the expression of NSUN2 have also been associated with multiple tumor types, including head and neck squamous cell carcinomas and breast, bladder, skin, colon, ovarian, esophagus, gallbladder, and gastric cancers [182,186,196,197,198,199]. In one study, the high expression of NSUN2 was verified to regulate the fate of tumor suppressor and oncogene transcripts, thereby promoting proliferation [212]. Recently, NSUN2 was also shown to regulate metastasis and drug resistance through the methylation of lncRNA in esophageal cancer [192]. In another study, the NSUN2-mediated aberrant methylation of the 3′UTR of oncogenic mRNA transcripts, such as those of heparin-binding growth factor, augmented their stability via a link to YBX1 [186]. NSUN3 directly binds to hnRNPK and transcription factors in leukemia to increase chemotherapeutic drug resistance [185]. NSUN4 is associated with an increased risk of prostate, ovarian, and breast cancer, and its overexpression is associated with liver cancer [195,196]. Additionally, in glioblastomas, the expression of NSUN5 is correlated with poor survival [213], while in low-grade gliomas, the high expression of NSUN7 is associated with a shorter survival time [197]. However, it is unclear whether the underlying molecular mechanisms of NSUN2 in other cancers can regulate mRNA modification.

Regarding tRNA methylation, it has been shown that NSUN2 is upregulated in skin tumor cell populations depending on the suitable deposition of m^5^C on tRNAs [174]. The loss of NSUN2 leads to the hypomethylation of tRNA, resulting in the accumulation of 5′-tRNA fragments [14,214]. Indeed, it is increasingly recognized that the biogenesis of tRNA fragments is mainly stimulated by environmental stressors, and their aberrant expression in cancer suggests a significant function in tumorigenesis [215]. Little is known about their functions, and studies have revealed that 5′-tRNAs can regulate protein translation in response to stress by controlling the binding of various translation initiation factors to the TIC [216,217]. Importantly, the lack of NSUN2 leaves cells in an undifferentiated and proliferation-inhibited state, which is required for tissue or cancer stem cell self-renewal [188,214,218]. Other studies have shown that cancer cells require the precise regulation of tRNA methylation, protein synthesis, and the biological function of tRNA fragments to maintain cellular responses and tumor volume [219]. Alterations in rRNA methylation are also associated with tumors. The expression level of NSUN5 is correlated with poor survival in glioblastoma patients. The epigenetic deletion of NSUN5 in glioma downregulates rRNA methylation levels and subsequently increases growth factor translation, making glioma cells resistant to the stress-related enzyme NAD(P)H quinone dehydrogenase 1 sensitive [213]. Thus, these findings underscore the importance of intracellular tRNA and rRNA methylation for the synthesis of various proteins and also suggest a role of RNA methylases in cancer development and progression.

#### 3.3.2. m^5^C Erasers in Cancer

Erasers of 5-hydroxymethylation have also been found to be altered in cancer. Mutations in or the altered expression of TET and ALKBH1 have been connected to certain malignancies. For instance, ALKBH1 and TET2 mutations are associated with myeloid and lymphoblastic leukemias [220,221], while TET1 is highly expressed in glioblastoma [222]. To the contrary, TET2 is downregulated in gliomas [200], whereas the epigenetic inhibition of TET3 may alter glioblastoma tumorigenesis [202]. While the mechanistic reasons are always related to DNA hydroxymethylation or demethylation deficiency, ALKBH1 and TET have also been indicated to oxidize m^5^C in RNA, suggesting that RNA hydroxymethylation deficiency is also associated with cancer.

### 3.4. Pseudouridine (Ψ)

Ψ, the C5 glycosidic isomer of uridine, was the first epigenetic modification to be discovered and is also one of the most abundant in RNA [223] (Figure 3E). Although Ψ was discovered decades ago, it was only recently that research began to reveal its biological functions (Table 5). Ψ was originally identified on tRNA and rRNA in yeast [224]; however, it has also been identified on different types of RNA, including small Cajal body-specific RNA, snRNA, tRNA, rRNA, miRNA, and lncRNA [225]. Most importantly, with current technological advances, further genome-wide analyses have successfully identified novel substrate mRNA molecules. The molecular function of Ψ in cancer is also becoming increasingly understood. The function of Ψ is mainly achieved through two important and different pathways: RNA-independent and RNA-dependent Ψ action. The RNA-dependent mechanism is mediated by an RNA–protein complex of small ribonucleoproteins, which consists of a box H/ACA snoRNA and four core proteins: glycine-arginine rich protein 1, nucleolar protein 10, nonhistone protein 2, and dyskerin (also known as NAP57 or DKC1) [226]. RNA-independent Ψ is catalyzed by a single pseudouridine synthase (PUS), which does not require RNA template strands for substrate recognition and catalysis [227]. In eukaryotic cells, there are more than 14 different Ψ enzymes, 10 of which are members of the PUS family [228]. Each of them has specific catalytic targets, but they also share some common targets.

#### 3.4.1. RNA-Dependent Ψ Synthetases in Cancer

The main modified substrates of dyskerin are SnRNAs and rRNAs, which are also involved in the activation of the telomerase complex [223]. DKC1-inactivating mutations are associated with X-linked dyskeratosis congenita (X-DC) and thereby increase the risk of cancer development in patients with X-DC [244].

DKC1 gene mutations have been linked to various cancers, such as skin cancer [245], breast cancer [229], CRC [232], lung cancer [232], prostate cancer [237], head and neck cancers [234], glioma [233], and liver cancer [235], as well as hematological malignancies and bone marrow failure syndromes, including chronic lymphocytic leukemia and multiple myeloma [236,237]. Mechanistically, the downregulation of pseudoribosylated rRNA attenuates the internal ribosome entry-dependent translation of tumor suppressor genes, such as p27, p53, and suppressors of apoptosis (XIAP, BclXL) [238,246]. The enhanced expression of vascular endothelial growth factor is related to the knockdown of DKC1, leading to an increased risk of oncogenesis in cells [247]. Other recent studies have reported that the loss of snoRNA, which is modified by pseudouridine at specific rRNA residues, increases protein mistranslation in hepatocellular carcinoma cells, resulting in changes in the ribosomal elongation rate, tRNA selection efficiency, and translation efficiency and thus affecting cancer cell survival [248]. Another study of rRNA Ψ modification found a reduction in 1-methyl-3-α-amino-α-carboxyl-propyl pseudouridine (m^1^acp^3^Ψ) modification in CRC, which affected the normal interactions between ribosomal P-sites and tRNA, altering and attenuating the translation efficiency in cancer cells [249].

Conversely, other studies have suggested that dyskerin plays a carcinogenic role. For example, DKC1 expression levels, the pseudouridylation of rRNA, and reduced telomere length are associated with a better prognosis in breast cancer [250]. In gliomas, the high expression of DKC1 stimulates the increased expression of glioma growth regulators to promote glioma cell migration and progression [233]. The data thus far suggest that alterations in DKC1 expression or activity are strongly associated with cancer, but the exact mechanism may depend on the cell, tissue, or DKC1 substrate. Further studies are needed to explore whether DKC1 has oncogenic or tumor-suppressive effects.

#### 3.4.2. RNA-Independent Ψ Synthetases in Cancer

The function of DKC1 in the occurrence and development of cancer is more well-studied, but little is known about the function of other pseudouridase enzymes, and only a handful of studies have shown their activity or expression related to tumors. For example, studies have identified PUS1 as a co-activator of retinoic acid receptor γ and other nuclear receptors in melanoma cells and breast cancer that simultaneously catalyze uridine in noncoding RNAs to pseudouridine to ensure proper protein folding and function [239]. PUS10 is present only in eukaryotes, and the dysregulation of PUS10 induces apoptosis in prostate cancer cells by reducing their sensitivity to the tumor necrosis factor-related apoptosis-inducing ligand [242]. Genomic alterations in PUS10 are pointedly correlated with lung cancer risk, but further studies are needed to demonstrate whether they are dependent on pseudouridase activity [243]. PUS7 is underexpressed in myelodysplastic syndromes, which may ultimately lead to a high risk of the disease transforming into acute myeloid leukemia [240]. Mechanistically, one study demonstrated the deletion of PUS7 in human hematopoietic stem and progenitor cells and that embryonic stem cells reduced PUS7-mediated pseudouridylation in a specific class of tRNA-derived RNA fragments, resulting in significantly higher rates of protein synthesis and leading to severe differentiation and growth defects [241]. Studies using patient-derived cancers and in vivo model cells will help uncover the clinical implications of targeting tRNA fragments for PUS7 deletion or aberration. Thus, the important role of tRNA fragments in reprogramming translation has been demonstrated [251].

### 3.5. ac^4^C in Cancer

ac^4^C, a recently acknowledged epigenetic modification within mRNA (Figure 3D), has been described as a crucial regulator of mRNA translation efficiency and stability. NAT10, the only known “writer” protein of ac^4^C, is thought to have vital effects on tumor metastasis and tumorigenesis. ac^4^C is present in more than 4000 human transcriptome regions. In human cervical cancer cells, ac^4^C is mainly enriched in the coding sequence region and decreases sequentially from the 5′UTR to the 3′UTR region of the gene transcript [252]. The function and mechanism of ac^4^C in gene expression regulation in a variety of cancers is summarized in Table 6. The downregulation of NAT10 was shown to result in the reduced ac^4^C modification of mRNA in specific regions of bladder cancer cells, impairing the translational stability and efficiency of BCL9L, SOX4, and AKT1. The deletion of NAT10 was also shown to reduce tumor burden in bladder cancer xenograft and transgenic mouse models [253]. Another study found that ac^4^C was elevated in the urine of CRC patients and was positively correlated with Duke’s CRC stage but significantly decreased after the radical resection of CRC [254]. Similarly, ac^4^C is also highly expressed in liver cancer patients. High ac^4^C expression in patients is correlated with an advanced tumor stage, high tumor stemness, high *TP53* mutation rate, low stromal score, high immune score, and greater activation of the pathways of the DNA repair system and regulation of T-cell infiltration [255]. Other researchers have found that NAT10 plays a vital function in gastric cancer metastasis by regulating the writing pathway of the gastric cancer mRNA ac^4^C [256]. At the same time, some scholars have analyzed the comprehensive TCGA and Genotype Tissue Expression databases and found that the overexpression of NAT10 in multiple cancers is significantly associated with a poor prognosis when compared to normal tissues. Notably, NAT10 expression in liver cancer was positively correlated with immune infiltration, including CD4+ T cells, CD8+ T cells, B cells, etc. [257]. As mentioned above, ac^4^C plays a significant role in the development and occurrence of various cancers; however, as far as the current research is concerned, most of these studies focus only on clinical patients and data analysis. To date, only a few molecular mechanism experiments have been performed to elucidate the role of ac^4^C in the specific mechanisms of various cancers.

### 3.6. mRNA Modification and Translation Factors: Crosstalk between m^6^A and eIFs in Cancer

Interestingly, recent findings have shown a correlation between m^6^A modification and human translation initiation factors that regulate the development and occurrence of cancer. METTL3 interacts with eIF3h to promote translation by affecting its multimer conformation. METTL3 promotes oncogene translation and tumorigenesis through an mRNA loop mechanism; however, the deletion of eIF3h abolishes these functions. This ability is similar to eIF4G-PABPC1-mediated mRNA looping [62,63]. Studies have also reported that eIF4A/4B can target 50 Wilms’ tumor-associated protein (WTAP), an adaptor of the m^6^A RNA methyltransferase complex, and mRNA transcripts whose translation can be controlled by mTORC1 downstream kinase S6K promotion, thereby promoting the occurrence and development of tumors [258,259].

Additionally, the knockdown of METTL3 was shown to significantly inhibit the expression of translation initiation factors, including eIF (2B3, 3b, 3c, 3d, 4A1, and 5/5A), thereby inhibiting lung adenocarcinoma proliferation and reducing the invasive ability of cancer cells. It was also shown that m^6^A methylation is required during translation [260]. In vitro experiments have shown that the *eIF3d* gene inhibits prostate cancer progression by regulating translation, the cell cycle, drug response, and multiple signaling pathways at m^6^A levels [60]. After METTL3 knockout, the distribution of the translation initiation factor eIF3 core subunit eIF3b, the cytosolic m^7^G cap-binding protein eIF4e, and the nuclear m^7^G cap-binding protein CBP80 shift from heavier multimeric components to lighter submultimeric fractions, resulting in attenuated translation and inhibiting cancer cell survival, growth, and invasion [123].

Studies have elucidated that eIF4A3 upregulates the binding of circular RNA (circRNA) and WTAP, promoting the formation of WTAP/METTL3/METTL14 m^6^A methyltransferase in bladder cancer tissues and cell lines. The formation of complexes reduces the chemosensitivity of bladder cancer to chemotherapeutic agents [261]. METTL14 directly binds to eIF4G1 mRNA and reduces the stability of eIF4G1 RNA by mediating m^6^A modification, thereby regulating autophagy levels and effectively reducing the growth of oral squamous cell carcinoma [262]. METTL16 is an RNA methyltransferase primarily responsible for the deposition of N6-methyladenosine (m^6^A) in transcripts. In the cytosol, METTL16 directly interacts with eIF3a and b and rRNA through its Mtase domain to promote the assembly of the TIC. METTL16 is critical for hepatocellular carcinoma tumorigenesis [263].

Importantly, one study found that circRNAs are enriched in m^6^A motifs and drive translation under the action of eIF4G2 and the m^6^A reader YTHDF3 [264]. METTL3 triggers m^6^A mRNA methylation, which further regulates the MALAT1-miR-1914-3p-YAP axis by recruiting YTHDF1/3 and eIF3b to the TIC, thus increasing YAP mRNA translation and stability. The high expression of YAP enhances the induction of drug resistance and metastasis in non-small cell lung cancer [15]. Meanwhile, YTHDF1 negatively affects eIF3a and b through the m^6^A mechanism and reduces the proliferation and clone formation of Merkel cell carcinoma in vitro [265]. In another study, TCGA analysis identified that the m^6^A-regulating genes *YTHDF1* and *eIF3b* were upregulated in CRC and confirmed that they were significantly differentially expressed between CRC and normal tissues by immunohistochemistry [266]. YTHDF1 promotes ovarian cancer tumorigenesis and metastasis by binding to m^6^A-modified eIF3c mRNA and enhancing eIF3c translation in a m^6^A-dependent manner while promoting overall translational output [264]. eIF2α kinase 2 bridges YTHDF3 and eIF3a to recognize the 5′UTR of m^6^A-methylated RNA and enhances YTHDF3/eIF3a complex stability in oxaliplatin-resistant CRC cells [267]. Following FTO silencing, YTHDF2 captures m^6^A-containing eIF4G1 transcripts, resulting in mRNA degradation and reduced eIF4G1 protein expression, thereby promoting autophagy and reducing tumorigenesis [268]. Emerging studies have reported that circPDE5A blocks the WTAP-dependent m^6^A methylation of eIF3c mRNA by forming a circPDE5A-WTAP complex, ultimately disrupting the translation of eIF3c for prostate cancer cell migration and invasion [269]. circPVRL3 was also predicted to bind eIF4A3 based on the structure of the internal ribosome entry, m^6^A modification, and open reading frame. The downregulation of circPVRL3 can promote the proliferation of gastric cancer [270].

## 4. Conclusions and Perspectives

In conclusion, numerous studies have demonstrated the importance of various RNA modifications and translational regulatory mechanisms in cancer. RNA modification and translation initiation are critical for tumor cell survival and proliferation and the expression of oncogenic proteins, affecting tumorigenesis and progression. RNA modification is a crucial post-transcriptional regulator of the gene expression program, and translation initiation is the critical and rate-limiting step; both have a key impact on the translational control of gene expression. At the same time, translation (translation rate, translation inhibition or enhancement, translation abnormality, etc.) can be determined by translation initiation factors as well as different modifications at different sites on the RNA transcripts. Recently, an increasing number of studies have focused on the functional network of the epitranscriptome and translational control via metabolism, epigenetics, and chromatin remodeling, and have recognized that the abnormal deposition of RNA modifications in cancer cells leads to mistranslation and a wide range of functional alterations.

Many enzymes responsible for RNA modification and regulation are targets of current cancer treatments. RNA epitranscriptomics, the exploration of RNA modifications, is a popular frontier. With the development of technology, new RNA modifications (such as ac^4^C) have been identified in cancer. Their biological functions and connections with cancer have been described, which has also provided new insights into future research. Identifying more unknown RNA-modifying enzymes and RNA modifications and determining the oncogenic or tumor-suppressive effects of aberrant modifications will guide the search for precise molecular targets and provide a theoretical basis for developing effective therapies against special tumor classes or other complex diseases. However, there is still much work to be completed to fully understand the complex relationship between RNA modification and translation in the development of tumor-targeted therapeutic drugs and biomarkers. At present, studies on translation regulation and RNA modifications are only the tip of the iceberg, but their rapid development will comprehensively expand our understanding of cancer.

## Figures and Tables

**Table 1:** Functions of eIFs and subunits in translation initiation. eIF—eukaryotic translation initiation factor; mRNA—messenger RNA; GTP—guanosine triphosphate; GDP—guanosine diphosphate; GAP—GTPase activating protein; tRNA—transfer RNA; TC—ternary complex; PIC—preinitiation complex; ATP—adenosine triphosphate; m^7^G—7-methylguanosine; UTR—untranslated region; PABP—poly(A)-binding protein; ATPase—adenosine triphosphatase; GTPase—guanosine triphosphatase; Met-tRNA_i_^Met^—initiator tRNA.

Class	Factor	Interacting eIFs	Functions	References
eIF1	eIF1	eIF2, eIF3, eIF5	Selection initiation codon, constructs 40S ribosome for mRNA loading and stimulates scanning with eIF1A, promotes binding between eIF2 triplet complex and 40S ribosomal subunit, and blocks the premature hydrolysis of eIF2–GTP to eIF2–GDP by eIF5 GAP to stop downstream procedures.	[23,24]
	eIF1A	eIF5B	Selection initiation codon, constructs 40S ribosome for mRNA loading and promotes scanning with eIF1, promotes binding between eIF2 triplet complex and 40S ribosomal subunit, and collaborates with eIF1 to promote ribosomal scanning and initiation codon selection	[25,26]
eIF2	eIF2	eIF1, eIF2B, eIF3, eIF5	Binds and recruits Met-tRNA_i_^Met^ to 40S ribosome, assembles the eIF2–TC complex and transports it to the 40S ribosomal subunit, and promotes the binding of Met-tRNA_i_^Met^ to the 40S ribosomal subunits. The phosphorylation state of eIF2α regulates the speed of formation of the TC.	[24,27]
	eIF2B	eIF2	Activates the eIF2 factor by stimulating the release of guanosine diphosphate (GDP) and helps in the regeneration of eIF2–TC complex	[28]
eIF3	eIF3a-m	eIF1, eIF1A, eIF4G, eIF5	Framework that organizes the 43S PIC; promotes the binding of eIF1, eIF4G, and eIF5 to the 40S ribosomal subunit; stimulates the attachment of the 43S ribosomal subunit to the eIF2–TC and mRNA; and prevents premature binding between the 40S and 60S subunits.	[16,29,30,31,32]
eIF4	eIF4A	eIF4G, eIF4B	ATP-dependent RNA helicase, member of eIF4F cap-binding complex, unwinds secondary structure in 5′UTR of the mRNA.	[32]
	eIF4B	eIF4A, eIF3	Promotes helicase activity of eIF4A, RNA-binding protein.	[16,32]
	eIF4E	eIF4G	Binds to mRNA at 5′ m^7^G cap, member of eIF4F cap-binding complex, stimulates eIF4A helicase activity with eIF4G.	[16,32,33]
	eIF4G	eIF4E, eIF4A, eIF3	Member of eIF4F cap-binding complex; assists in the binding of eIF3, eIF4A, eIF4E, PABP, and mRNA; stimulates the helicase and ATPase activity of eIF4A.	[29,32,34]
	eIF4H	eIF4A	Enhances the RNA helicase activity of eIF4A (eIF4F), RNA-binding protein, homologous to the N-terminus activity of eIF4A.	[35]
eIF5	eIF5	eIF1, eIFA1, eIF2, eIF3	Activates eIFs by its GTPase nature.	[36,37]
	eIF5B	eIF1A	Ribosome-dependent GTPase, responsible for connection between ribosomal subunits.	[25]

**Table 2:** Role of m^6^A in cancer. YTHDC1—YTH domain-containing 1; YTHDC2—YTH domain-containing 2; METTL3—methyltransferase-like 3; ALKBH5—alkB homolog 5; FTO—fat mass and obesity-associated gene; AML—acute myeloid leukemia; HCC—hepatocellular carcinoma; GSC—glioma stem cells.

Type	Factor	Tumor	Role	Targets	References
Writers	METTL3	AML	Oncogene	c-MYC, BCL2, PTEN	[114,115]
		Bladder cancer	Oncogene	CPCP1, NF-κB, MYC	[116]
		Breast cancer	Oncogene	HBXIP, let-7g	[117]
		Colon cancer	Oncogene	SPRED2, MAPK	[118]
		Endometrial cancer	Suppressor	AKT, PHLPP2, mTORC2	[119]
		Glioblastoma	Suppressor	ADAM19, EPHA3, KLF4, CDKN2A, BRCA2, TP53I11	[12]
		Glioblastoma	Oncogene	BCL-X, NCOR2	[120]
		Liver cancer	Oncogene	SOCS2, YTHDF2	[121]
		Colon cancer	Oncogene	IGF2BP2, SOX2	[122]
		Lung cancer	Oncogene	EGFR, TAZ, MAPKAPK2, DNMT3A, BRD4	[63,123]
		Melanoma	Oncogene	c-Met, p-Akt	[124]
		Osteosarcoma	Oncogene	LEF1, Wnt/β-catenin	[125]
		Ovarian cancer	Oncogene	AXL, EMT	[126]
		Prostate cancer	Oncogene	GLI1, ITGB1	[127,128]
		Renal cell carcinoma	Suppressor	CAM, Wnt/β-catenin, EMT), PI3K-Akt-mTOR	[129,130]
		Leukemia	Oncogene	c-MYC, BCL2, and PTEN	[131]
	METTL14	AML	Oncogene	MYB, MYC, SPI1	[115]
		Bladder cancer	Suppressor	Notch1	[132]
		Colon cancer	Suppressor	YAP, lncRNA XIST	[133,134]
		Endometrial cancer	Suppressor	AKT, PHLPP2, mTORC2	[119]
		Renal cell carcinoma	Oncogene	ATP-P2RX6, p-ERK1/2/MMP9	[135]
		Pancreatic cancer	Oncogene	PERP	[136]
Erasers	FTO	AML	Oncogene	ASB2, RARA	[137]
		Bladder cancer	Oncogene	MALAT1, MAL2	[138]
		Breast cancer	Oncogene	BNIP3	[139]
		Cervical cancer		E2F1, Myc	[140]
		Glioblastoma	Suppressor	ADAM19	[12]
		HCC	Oncogene	SOX2, KLF4, NANOG	[141]
		Lung cancer	Oncogene	MZF1	[142]
		Melanoma	Oncogene	PD-1, CXCR4, SOX10	[143]
		Pancreatic cancer	Oncogene	MYC, bHLH	[144]
	ALKBH5	AML	Oncogene	TACC3	[145]
		Breast cancer	Oncogene	NANOG	[146]
		Glioblastoma	Oncogene	FOXM1	[147]
		Gastric cancer	Oncogene	NEAT1	[148]
		Lung cancer	Suppressor	miR-107/LATS2	[149]
		Osteosarcoma	Oncogene	PVT1	[150]
		Ovarian cancer	Oncogene	NANOG, TLR4, NF-κB	[151]
		Pancreatic cancer	Oncogene	WIF-1	[152]
Readers	YTHDC2	Colon cancer	Oncogene	HIF-1α	[153]
	YTHDF1	Colon cancer	Oncogene	Wnt/β-catenin	[154]
		Melanoma	Suppressor	HINT2	[155]
		Ovarian cancer	Oncogene	eIF3c	[58]
	YTHDF2	AML	Oncogene	Tnfrsf2	[156]
		HCC	Suppressor	EGFR	[157]
		Lung cancer	Oncogene	6PGD	[158]

**Table 3:** Role of m^6^Am in cancer. FTO—fat mass and obesity-associated gene; PCIF1—phosphorylated CTD-interacting factor 1.

Type	Factor	Tumor	Role	Targets	References
Writers	PCIF1	Glioma	Suppressor	Unknown	[177]
Erasers	FTO	Colorectal cancer	Oncogene	Unknown	[175]
Readers	Unknown	Unknown	Unknown	Unknown	

**Table 4:** Role of the aberrant deposition of m^5^C in cancer. NSUN—NOP2/Sun; TET1—tet-eleven translocation 1; ALKBH1—alkB homolog 1; YBX1—Y-box binding protein 1; AML—acute myeloid leukemia; ALL—acute lymphoblastic leukemia; HCC—hepatocellular carcinoma.

Type	Factor	Tumor	Role	Targets	References
Writers	NSUN1/NOP2/p120	Breast cancer	Oncogene	Myc	[182]
		Lung cancer	Oncogene	Unknown	[183]
		Prostate cancer	Oncogene	Unknown	[184]
	NSUN2	Leukemia	Oncogene	hnRNPK, TF, GATA1, SPI1	[185]
		Bladder cancer	Oncogene	HDGF	[186]
		Skin, breast, and colon cancer	Oncogene	Unknown	[187]
		Squamous cell carcinoma	Oncogene	Unknown	[188]
		Gallbladder carcinoma	Oncogene	RPL6	[189]
		Gastric cancer	Oncogene	CDKN1C	[190]
		Head and neck squamous carcinoma	Oncogene	Unknown	[191]
		Esophageal squamous cell carcinoma	Oncogene	NMR, BPTF	[192]
		Ovarian cancer	Oncogene	Unknown	[193]
		Several cancer types	Oncogene	Aurora-B	[194]
	NSUN3	Leukemia	Oncogene	hnRNPK, TF, GATA1, SPI1	[185]
	NSUN4	Breast, ovarian, and prostate cancer	Oncogene	Unknown	[195]
		HCC	Oncogene	Unknown	[196]
	NSUN5	Glioblastoma	Oncogene	Unknown	[197]
	DNMT2	Somatic cancer	Oncogene	R371H, G155 V	[198]
		Leukemia	Oncogene	hnRNPK, TF, GATA1, SPI1	[185]
Erasers	TET1	Glioblastoma	Oncogene	miRNA-339-5p	[199]
	TET2	Glioblastoma	Oncogene	Unknown	[200]
		AML	Oncogene	Unknown	[201]
	TET3	Glioblastoma	Oncogene	Unknown	[202]
	ALKBH1	ALL	Oncogene	Unknown	[147]
		Gastric cancer	Oncogene	Unknown	[203]
Readers	YBX1	Bladder cancer	Oncogene	MDR-1	[204]
		Breast cancer	Oncogene	ESR1-FOXA1	[205]
		Several cancer types	Oncogene	AKT, p70S6K, p90RSK	[206]
	ALYREF	HCC	Oncogene	Unknown	[196]
		Oral squamous cell carcinoma	Oncogene	Unknown	[207]
		Several cancer types	Oncogene	Unknown	[208]

**Table 5:** Role of pseudouridylases in cancer. DKC1—dyskerin pseudouridine synthase 1; PUS1—pseudouridylase synthase 1; AML—acute myeloid leukemia; CLL—chronic lymphocytic leukemia; HCC—hepatocellular carcinoma.

Type	Factor	Tumor	Role	Targets	References
Writer	DKC1	Breast cancer	Oncogene	Unknown	[229]
		CLL	Oncogene	Unknown	[230]
		Lung cancer	Oncogene	TERC	[231]
		Colorectal cancer	Oncogene	ALT-TMM	[232]
		Glioblastoma	Oncogene	N-cadherin, HIF-1α, MMP2	[233]
		Head and neck cancer	Oncogene	Unknown	[234]
		HCC	Oncogene	MKI67, MYC	[235]
		Multiple myeloma	Oncogene	Unknown	[236]
		Prostate cancer	Oncogene	H/ACA snoRNAs	[237]
		Pituitary cancer	Suppressor	p27	[238]
	PUS1	Melanoma and breast cancer	Oncogene	SRA1, RARγ, ER	[239]
	PUS7	Myelodysplastic syndromes/AML	Oncogene	Unknown	[240,241]
	PUS10	Prostate cancer	Oncogene	TRAIL	[242]
		Lung cancer	Oncogene	Unknown	[243]
Erasers	Unknown	Unknown	Unknown	Unknown	
Readers	Unknown	Unknown	Unknown	Unknown	

**Table 6:** Role of ac^4^C in cancer. HNSCC—head and neck squamous cell carcinoma; KRPCC—kidney renal papillary cell carcinoma.

Type	Factor	Tumor	Role	Targets	References
Writer	NAT10	Bladder cancer	Oncogene	BCL9L, SOX4, AKT1	[253]
		Colorectal cancer	Overexpress	Unknown	[254]
		Liver cancer	Overexpress	Unknown	[255]
		Gastric cancer	Metastasis	COL5A1	[256]
		Adrenocortical carcinoma	Overexpress	Unknown	[257]
		HNSCC	Overexpress	Unknown	
		KRPCC	Overexpress	Unknown	
		Pheochromocytoma	Overexpress	Unknown	
		Paraganglioma	Overexpress	Unknown	
Erasers	Unknown	Unknown	Unknown	Unknown	
Readers	Unknown	Unknown	Unknown	Unknown	

## Data Availability

All data are contained within this article and available from the corresponding author on reasonable request.
